# NEONATAL TINEA CORPORIS

**DOI:** 10.4103/0019-5154.62741

**Published:** 2010

**Authors:** Ashok Kumar Khare, Lalit Kumar Gupta, Asit Mittal, C M Kuldeep, Anshu Goyal

**Affiliations:** *From the Department of Dermatology, Venereology & Leprology, R.N.T. Medical College, Udaipur - 313 001, Rajasthan, India.*

**Figure 1:**
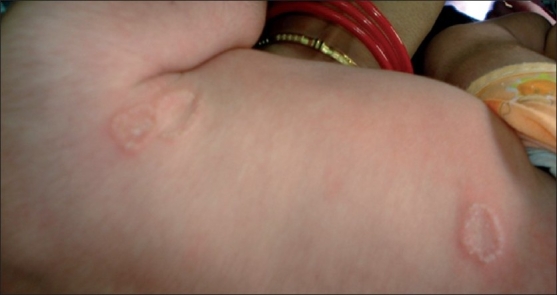
Typical lesions of tinea corporis on trunk

Sir,

Fungal infection of skin in the full-term new born is uncommon except for candidal vesicopustular diaper rash and thrush.[1] We report a 20-day-old full-term neonate presenting with 10 day history of multiple, scaly, erythematous, annular plaques with raised margins typical Of tinea corporis over trunk [Figure 1] and extremities. None of the family members showed any evidence of dermatophytosis. There was no history of keeping pets in the family. KOH examination showed numerous septate branching hyphae typical of dermatophytes. Culture on Sabouraud's dextrose agar media grew *Trichophyton rubrum*. The baby was treated with 1% topical clotrimazole cream with complete resolution of lesions in 2 weeks.

The cases of neonatal tinea are rarely encountered in dermatology clinics. This could possibly be due to high sebum secretion rates in neonates.[2] Sebum has been shown to have antibacterial and antifungal properties.[2]

Although neonatal tinea is rare, cases[3–5] have been reported occasionally. The appearance of lesions at the age of 10 days in our case was interesting. The incubation period of tinea infection varies from 1 to 3 weeks. However, a shorter incubation period has also been shown experimentally.[6] The source of infection could not be traced in this case. Asymptomatic family member or unidentified contact as a carrier of trichophyton rubrum cannot entirely be ruled out. Such a carrier state has been reported.[7]
